# Bedeutung von „avoidance“ und „endurance“ beim Post-COVID-Syndrom: Sind dysfunktionale Muster veränderbar?

**DOI:** 10.1007/s00482-025-00887-5

**Published:** 2025-06-20

**Authors:** Alexa Kupferschmitt, Christoph Herrmann, Michael Jöbges, Stefan Kelm, Gerhard Sütfels, Thomas H. Loew, Monika Hasenbring, Volker Köllner

**Affiliations:** 1 Forschungsgruppe Psychosomatische Rehabilitation, Abteilung für Psychosomatische Medizin, Zentrum für Innere Medizin und Dermatologie, Charité – Universitätsmedizin Berlin, 10098 Berlin, Deutschland; 2 Abteilung für Psychosomatische Medizin, Rehabilitationszentrum Seehof, Lichterfelder Allee 55, 14513 Teltow, Deutschland; 3 Abteilung für Psychosomatische Medizin, Universitätsklinikum Regensburg, Rilkestraße 39, 93049 Regensburg, Deutschland; 4 Kliniken Schmieder, Auf dem Berg 1, 78262 Gailingen, Deutschland; 5 Kliniken Schmieder, Eichhornstraße 68, 78464 Konstanz, Deutschland; 6 Westerwaldklinik Waldbreitbach, Buchenstraße 6, 56588 Waldbreitbach, Deutschland; 7 Reha-Zentrum Todtmoos, Klinikum Wehrawald, Schwarzenbacher Straße 4, 79682 Todtmoos, Deutschland; 8 Abteilung für Medizinische Psychologie und Soziologie, Fakultät für Medizin, Ruhr-Universität Bochum, 44781 Bochum, Deutschland

**Keywords:** Avoidance-Endurance-Modell, Avoidance-Endurance Questionnaire, Durchhaltemuster, Vermeidungsverhalten, Post-COVID-Rehabilitation, Avoidance endurance model, Avoidance endurance questionnaire, Persistence pattern, Avoidance behavior, Post-COVID rehabilitation

## Abstract

**Hintergrund:**

Bei der Chronifizierung von Schmerz ist die Bedeutung dysfunktionaler Copingstrategien gut belegt. Bewährt hat sich hier das Avoidance-Endurance-Modell (AEM), das sich nach ersten klinischen Erfahrungen auch beim Post-COVID-Syndrom (PCS; *COVID* „coronavirus disease“) gut eignet, um dysfunktionales Krankheitsverhalten abzubilden. Ziel der vorliegenden Studie ist es nachzuweisen, welche Muster bei Patienten mit PCS wie häufig vorkommen und ob sie sich im Rahmen einer multimodalen Rehabilitation (Reha) verändern.

**Methode:**

Im Rahmen der Multicenterstudie PoCoRe wurden *N* = 481 PCS-Rehabilitanden hinsichtlich des Krankheitsverhaltens nach dem AEM zu Reha-Beginn und -Ende untersucht. Häufigkeitsanalysen, Chi^2^-Test und Sankey-Diagramm kamen zur Anwendung.

**Ergebnisse:**

Zu Reha-Beginn wiesen etwa 81,8 % der PCS-Patienten ein dysfunktionales Krankheitsverhalten auf, wobei hiervon 57,7 % dysfunktionale Durchhalter waren („distress-endurance response“); bei 24,1 % lag „fear-avoidance“ vor, bei 10,0 % „eustress-endurance“ und bei 10,5 % eine „adaptive response“. Über den Reha-Verlauf verschoben sich die Verhaltensmuster um 8,2 % hin zur „adaptive response“, und um 12,7 % zu „eustress-endurance“, hauptsächlich von den vormaligen Distress-endurance-Typen kommend (−16,7 %). „Fear-avoidance“ nahm um etwa 4,8 % ab. Innerhalb der einzelnen AEM-Reaktionsmuster können sich sowohl dysfunktionale Muster zu funktionalen Mustern als auch funktionale Muster zu dysfunktionalen Mustern verändern.

**Schlussfolgerung:**

Das deutliche Überwiegen der dysfunktionalen Muster in dieser hoch chronifizierten Stichprobe spricht dafür, dass das Avoidance-Endurance-Konzept auch bei der Chronifizierung von Fatigue im Rahmen des PCS relevant ist. Im Gegensatz zu Patienten mit chronischem Schmerz überwiegen hier aber deutlich die Endurance-Muster. Es zeigte sich eine deutliche Verschiebung hin zum funktionalen Muster während der Reha, was für die Modifizierbarkeit spricht. Etwa 10–15 % der Patienten entwickelten sich jedoch in eine ungünstige Richtung, was bei der Behandlungsplanung berücksichtigt und weiter untersucht werden sollte.

**Zusatzmaterial online:**

Die Online-Version dieses Artikels (10.1007/s00482-025-00887-5) enthält eine weitere Tabelle mit deskriptiven Statistiken der Gesamtstichprobe und der einzelnen Indikationen.

## Abstract (en)

**Background:**

The importance of dysfunctional coping strategies in the chronification of pain is well documented. The avoidance endurance model (AEM) has proven to be well-suited which, according to initial clinical experiences, is also well-suited to depicting dysfunctional illness behavior in post-COVID syndrome (PCS, *COVID* coronavirus disease). The aim of this study is to demonstrate which patterns occur and how frequently in PCS patients and whether they change in the context of multimodal rehabilitation.

**Method:**

As part of the PoCoRe multicenter study, *N* = 481 PCS rehabilitation patients were examined with respect to illness behavior according to the AEM at the start and end of rehabilitation. Frequency analyses, χ^2^-tests and Sankey diagrams were used.

**Results:**

At the start of rehabilitation around 81.8% of PCS patients exhibited dysfunctional illness behavior, of which 57.7% were dysfunctional endurers (distress endurance response), in 24.1% fear avoidance was present, in 10.0% eustress endurance and in 8.2% an adaptive response. Over the course of rehabilitation the behavioral patterns shifted by 8.8% towards adaptive response and by 12.7% to eustress endurance, which mainly comes from the former distress endurance types (−6.7%). Fear avoidance decreased by approx. 4.8%. Within the individual AEM reaction patterns, dysfunctional patterns can change in favor of functional patterns as well as functional patterns in favor of dysfunctional patterns.

**Conclusion:**

The clear predominance of dysfunctional patterns in this highly chronified sample suggests that the avoidance endurance concept is also relevant in the chronification of fatigue in PCS. In contrast to chronic pain patients, however, the endurance patterns clearly predominate here. There was a clear shift towards the functional pattern during rehabilitation, which speaks in favor of modifiability; however, around 10–15% of patients developed in an unfavorable direction, which should be taken into account in treatment planning and investigated further.

## Einführung

Die Art und Weise, wie eine Person mit chronischen Beschwerden, beispielsweise mit Schmerzen, gewohnheitsmäßig an Aktivitäten herangeht, kann einen Einfluss sowohl auf die Chronifizierung als auch auf die Beeinträchtigungen hinsichtlich Aktivität und Teilhabe haben [1, 2]. Die Annahme, dass das Vermeiden von Alltagsaktivitäten mit einer funktionellen Verschlechterung und einem höheren Chronifizierungsrisiko verbunden ist, ist empirisch gut belegt [3, 4]. Im Kontext von Fatigue bei Virusfolgeerkrankungen wie dem Post-COVID-19-Syndrom (PCS; *COVID-19* „coronavirus disease 2019“) gilt diese Annahme auch für Überaktivität (Aktivität, die die Symptome erheblich verschlimmert; [5]).

Im Zuge der COVID-19-Pandemie wird kurz- und langfristig eine Zunahme chronischer Schmerzen erwartet

Kernsymptome des PCS sind neben Fatigue kognitive Störungen, Atembeschwerden und insbesondere auch Schmerzen. Diese Symptome persistieren nach einer Severe-acute-respiratory-syndrome-coronavirus-type-2(SARS-CoV-2)-Infektion länger als 12 Wochen und führen zu relevanten Einschränkungen im Alltag [6]. Bei der Schmerzsymptomatik kann es sich um einen diffusen Ganzkörperschmerz handeln; der Schmerz kann aber auch spezifischer sein und beispielsweise gezielt Arme, Beine und Gelenke betreffen. Studien zeigen, dass nach einer COVID-19-Erkrankung vermehrt Fälle von lang anhaltenden Kopfschmerzen und Migräne [7], Brustschmerzen, Hodenschmerzen und chronischen Schmerzen auftreten [8]. Auch wird als Folge der COVID-19-Pandemie sowohl kurz- als auch langfristig eine Zunahme chronischer Schmerzen erwartet [9]. Untersuchungsergebnissen [10] zufolge liegt bei 56,3 % der Patienten mit PCS ein Schmerzsyndrom vor. Drei Monate nach der akuten COVID-19-Erkrankung wurden in 40,55 % der Fälle Myalgien, in 39,18 % Gelenkschmerzen, in 31,62 % Rückenschmerzen und in 24,74 % Schmerzen im unteren Rücken festgestellt. Nach 6 Monaten wurden bei 18,59 % der Patienten weiterhin Gelenkschmerzen, bei 15,09 % Myalgien, bei 14,39 % Rückenschmerzen und bei 11,23 % Schmerzen im unteren Rückenbereich beobachtet. In 50,8 % der Fälle berichteten die Patienten über neu aufgetretene Schmerzen, von denen 38,5 % einen mittleren Schweregrad (≥ 3 Punkte auf der visuellen Analogskala) aufwiesen [11]. In der Gesamtbevölkerung sind Kopf- oder Muskelschmerzen ein häufiges Symptom; für PCS-Patienten liegt das relative adjustierte Risiko, 12 Monate nach einer COVID-19-Erkrankung im Vergleich zu nicht Erkrankten unter Schmerzen zu leiden, bei 6,7 (95 %-Konfidenzintervall [Kl] 3,6–12,6) für Brustschmerzen, 1,8 (95 %-Kl 1,2–2,6) für Kopfschmerzen und 1,7 für Gelenk- und Muskelschmerzen (95 %-Kl 0,8–3,4; [12]).

Trotz des deutlich erhöhten Risikos ist die Angabe persistierender Schmerzen nicht gleichzusetzen mit Beeinträchtigungen im Alltag [7]. Relevante Beeinträchtigungen wie fortbestehende Arbeitsunfähigkeit werden bei bis zu 10 % der Betroffenen 7 Monate [13] bzw. 12 Monate nach der akuten Infektion berichtet [14]. Eine noch offene und dringend zu klärende Frage betrifft die Prädiktoren und die pathophysiologischen Mechanismen der verschiedenen Schmerzsyndrome nach SARS-CoV-2-Infektion (vgl. [7]).

Das Avoidance-endurance-Modell (AEM; [15]) ist ein umfangreich validiertes psychosoziales Modell, das sich für die Abbildung von Krankheitsverhalten eignet, welches auf Symptomatiken, wie chronische Schmerzen, modulierend wirken kann. Das AEM unterscheidet vier Schmerzbewältigungsmuster:

- Ängstliches Vermeiden („fear-avoidance response“ [FAR])
- Eustress-Durchhaltemuster („eustress-endurance response“ [EER])
- Distress-Durchhaltemuster („distress endurance response“ [DER])
- Adaptive Schmerzreaktion („adaptive response“ [AR])

Die Reaktionsmuster FAR und DER stellen maladaptive Schmerzreaktions- oder Schmerzbewältigungsmuster dar. Dabei spielen sowohl Angst vor Schmerzen und Vermeidungsverhalten als auch Gedankenunterdrückung und durchhaltendes Verhalten eine vermittelnde Rolle bei der Aufrechterhaltung von Schmerzen und sind dadurch ein Risikofaktor für die Chronifizierung. Eine adaptive Schmerzverarbeitung (AR), die durch einen flexiblen Wechsel zwischen Belastung (z. B. Berufsbelastung) und Entspannung (z. B. Erholung/Pausen) gekennzeichnet ist, trägt zur Schmerzreduktion bei. Inwiefern EER einen funktionalen oder dysfunktionalen Einfluss hat, ist noch nicht abschließend geklärt.

Das AEM wurde neben Patienten mit akuten/subakuten und chronischen Kreuzschmerzen [16, 17] auch an Patienten mit leichten bis moderaten Gehirnerschütterungssymptomen evaluiert [18]. Patienten litten noch Wochen danach an Symptomen wie Kopfschmerzen, Übelkeit und Müdigkeit und es konnte das Vorherrschen des FAR-Musters nachgewiesen werden. Hulme et al. [19] zeigten in einer systematischen Übersichtsarbeit, dass die kognitiven und verhaltensbezogenen Reaktionen auf einen akuten Infekt prädiktive Faktoren für ein postinfektiöses Müdigkeitssyndrom sind, Beispiele für diese Reaktionen sind negativer Perfektionismus, eine geringe Kontrollüberzeugung und ein „Alles-oder-nichts“-Verhaltensmuster, das heißt eine übertriebene Aktivität an Tagen, an denen es Patienten besser geht, um sich dann längere Zeit wieder auszuruhen. Im Sinne des AEM könnte man auch hier von durchhaltenden, katastrophisierenden und vermeidenden Reaktionen sprechen. Basierend auf dem AEM stützen erste Befunde die Rolle von FAR und von DER auch bei Patienten mit einem PCS [20].

Wenig ist bisher bekannt über Häufigkeit und Veränderbarkeit der AEM-Muster im Rahmen chronischer Erkrankungen. Eine genauere Kenntnis erleichtert jedoch das Vorhalten gezielterer kognitiv-behavioraler Therapiebausteine innerhalb einer multidisziplinären Versorgung, so auch in der Rehabilitation (Reha). Zur Auftretenshäufigkeit lässt sich für die Schmerzforschung sagen, dass im Reha-Bereich die Gruppe der Patienten mit adaptiver Verarbeitung einen Anteil zwischen 15 und 25 % ausmacht [17, 21]. Eine erste Studie zur Veränderbarkeit der AEM-Muster ergab vor Reha eine Zweiteilung in Patienten mit einem vermeidenden/Pacing-basierten Verhalten (43 %) und solche mit Überaktivität (57 %; [22]). Nach Beendigung eines 12-stündigen kognitiv-behavioralen Gruppenprogramms zeigte sich eine Ausdifferenzierung in vier Subgruppen:

- 27 % mit adaptiver Verarbeitung
- 19 % mit vermeidendem Verhalten
- 24 % mit Überaktivität
- 30 % mit Alles-oder-nichts-Verhalten

Ein Anteil von 30 % der vermeidenden Gruppe hatte nach Therapie das Verhalten in Richtung adaptiv verbessert, 25 % waren es in der Gruppe der anfangs Überaktiven. Für Patienten mit einem PCS stellten sich uns somit folgende Fragen:

- Wie häufig liegen die einzelnen AEM-Reaktionsmuster zu Reha-Beginn und zu Reha-Ende vor?
- Unterscheiden sich die PCS-Reha-Indikationen hinsichtlich der Verteilung der AEM-Reaktionsmuster?
- Veränderungen der AEM-Reaktionsmuster über den Reha-Verlauf: Gibt es Wanderungsbewegungen hinsichtlich der Zugehörigkeit zu den AEM-Reaktionsmustern?

## Methoden

### Vorgehensweise und Stichprobe

Im Rahmen der PoCoRe-Multicenterstudie [23] wurden die oben genannten Fragestellungen bearbeitet. Die prospektive Kohortenstudie basiert auf standardisierten Untersuchungen, wobei das Krankheitsverhalten mittels Avoidance-Endurance Questionnaire (AEQ) erfasst wurde. Die Datenerhebung fand zwischen März 2022 und August 2023 zu Beginn und zu Ende der stationären Reha (Durchschnittsdauer 5 Wochen) in vier Indikationen deutscher Reha-Kliniken statt, die ein Post-COVID-Konzept umgesetzt haben: duale Reha (Psychopneumologie, Psychokardiologie), Psychosomatik, Neurologie und Pneumologie. Es wurden insgesamt *N* = 478 Patienten mit PCS eingeschlossen. Die Genehmigung der Forschung erteilte die Ethikkommission des Zentrums für Klinische Forschung des Universitätsklinikums Regensburg (Kennung 22-2814-101) im Februar 2022.

### Messinstrumente

**Bewältigungsstile – AEQ. **Der AEQ ist ein auf dem AEM basierender, validierter Selbstauskunftsfragebogen, mit dem emotionale, kognitive und verhaltensbezogene Reaktionen auf Schmerzen, die zur Chronifizierung von Symptomen beitragen können, erfasst werden [24]. Er umfasst 49 Items auf zwei affektiven Skalen (Angst/Depressivität [ADS], positive Stimmung trotz Fatigue [PMS]), drei kognitiven Skalen (Hilf‑/Hoffnungslosigkeit [HHS], Katastrophisieren [CTS], Gedankenunterdrückung [TSS]) sowie vier verhaltensbezogenen Skalen (Meiden sozialer Aktivitäten [ASAS], Meiden körperlicher Aktivitäten [APAS], Humor/Ablenkung [HDS], „pain persistence“ [PPS]). Die Summe von HDS und PPS geht in einen Gesamtscore der „behavioral endurance“ (BES) ein. Das Antwortformat ist eine 7‑stufige Skala von 0 = nie bis 6 = jedes Mal. Eine ausreichende bis exzellente Reliabilität (Cronbachs α: 0,76–0,91) wurde für alle Subskalen nachgewiesen; die konvergente, divergente sowie kriterienbezogene Validität sind gut belegt (unter anderem [17, 24]). In der vorliegenden Studie wurde eine modifizierte Version des AEQ vorgegeben, bei der die Reaktionen auf Fatigue/Erschöpfung bezogen werden.

Die AEM-Reaktionsmuster der Patienten wurden nach Hasenbring et al. [16] klassifiziert. Die Klassifizierung basiert auf den Beck-Depression-Inventory-II(BDI-II)-Scores der Probanden sowie auf ihren mittleren Scores auf den AEQ-Skalen Gedankenunterdrückung (TSS) und Durchhalteverhalten (BES). Für BES wurde die Antwortskala „starke Fatigue/Erschöpfung“ eingesetzt. In der vorliegenden Studie wurde anstelle des BDI-II der Patient Health Questionnaire‑9 (PHQ-9) verwendet. In der Fassung von Hasenbring et al. wurde als Cut-off ein BDI-II-Wert von 9 angegeben, was einer minimalen Depression entspricht. Daher wurde in der vorliegenden Studie im PHQ‑9 ein Cut-off von 10 festgelegt, was ebenfalls einer leichten/unterschwelligen depressiven Störung entspricht [25].

- *FAR: *Subskala: TSS < 3,0 und BES < 3,0 und PHQ-9 ≥ 10
- *DER: *Subskala: TSS ≥ 3,0 und/oder BES ≥ 3,0 und PHQ-9 ≥ 10
- *EER: *Subskala: TSS ≥ 3,0 und/oder BES ≥ 3,0 und PHQ-9 < 10
- *AR: *Subskala: TSS < 3,0 und BES < 3,0 und PHQ-9 < 10

### Statistische Analyse

Die statistische Auswertung wurde mit dem Statistikprogramm SPSS 28 für Windows durchgeführt. Deskriptive Statistiken zur Stichprobenbeschreibung, Häufigkeitsanalysen zur Bestimmung der Anzahl der AEM-Reaktionsmuster, Chi^2^-Test zur Überprüfung signifikanter Unterschiede zwischen den Reha-Indikationen. Wenn Daten zu kontinuierlichen Variablen in weniger als 10 % aller Fälle fehlten, wurden diese imputiert (Mittelwert der Gesamtstichprobe); wenn Daten in mehr als 10 % aller Fälle fehlten, wurden Probanden mit fehlenden Werten von der jeweiligen Analyse ausgeschlossen. Die Patienten wurden bei Aufnahme in die vier AEM-Reaktionsmuster gruppiert und anschließend jede einzelne Gruppe dahingehend untersucht, ob der bei Aufnahme vorliegende AEQ-Typ bis zur Entlassung beibehalten wurde oder ob es signifikante Wechsel gab und wenn ja wohin. Zur grafischen Darstellung der Wechselbewegungen der AEM-Reaktionsmuster wurde ein Sankey-Diagramm erstellt. Hierfür wurden die Patientendaten verwendet, die sowohl bei Aufnahme als auch bei Entlassung das AE-Muster bestimmen ließen (n = 351).

## Ergebnisse

### Stichprobenbeschreibung

Im Zeitraum von März 2022 bis August 2023 wurden insgesamt 478 PCS-Patienten der interdisziplinären, PCS-spezifischen Reha mittels AEQ untersucht. In dieser Stichprobe waren 69,9 % der PCS-Patienten weiblich, das Durchschnittsalter betrug 48,30 Jahre (Standardabweichung [SD] = 9,92 Jahre). 19,3 % der PCS-Patienten waren seit 2020 erkrankt, 30,5 % seit 2021 und 48,7 % seit 2022. Beruflich waren 63,2 % der PCS-Patienten zu Reha-Beginn krankgeschrieben und dies zu 78,0 % auch schon länger als 6 Monate. Ein Anteil von 63,2 % der PCS-Patienten wurde arbeitsunfähig entlassen, wobei 61,5 % bzw. 71,6 % eine positive sozialmedizinische Prognose für den Bezugsberuf bzw. für den allgemeinen Arbeitsmarkt erhielten. Die Beschreibung der Gesamtstichprobe findet sich in Tab. 1. Die Stichproben der Reha-Indikationen/-Einrichtungen unterschieden sich teilweise in einzelnen Bereichen signifikant voneinander (Tab. S1 im Online-Zusatzmaterial). In der pneumologischen PCS-Reha wurden häufiger Frauen behandelt (79,7 %) als in der Psychosomatik (67,7 %) oder Neurologie (64,1 %). Psychosomatische PCS-Patienten waren deutlich jünger (M = 41,84 Jahre, SD = 8,48 Jahre) als PCS-Patienten in den restlichen Reha-Indikationen (M = 47,10 Jahre bis M = 51,77 Jahre). Auch hinsichtlich der Arbeitsfähigkeit bei Reha-Beginn lagen Unterschiede vor. Psychosomatische PCS-Patienten wurden am häufigsten arbeitsunfähig aufgenommen (68,6 %), pneumologische PCS-Patienten waren deutlich seltener arbeitsunfähig (46,9 %). Hinsichtlich der Dauer der Krankschreibung vor Reha-Beginn waren psychosomatische PCS-Patienten wiederum signifikant seltener über 6 Monate krankgeschrieben als die restlichen PCS-Patienten.

**Tab. 1:** Stichprobenbeschreibung* (N* *=* *478)*

*Demografische Merkmale*	
Altersdurchschnitt (Jahre)	48,30 (9,92)
Geschlecht (% weiblich)	69,9 %
*Berufliche Merkmale*	
Arbeitsunfähigkeit bei Aufnahme	60,4 %
Arbeitsunfähigkeitszeiten vor Rehabilitationsbeginn	15,4 % < 3 Monaten
	6,6 % 3–6 Monate
	78,0 % > 6 Monate
*Sozialmedizinische Beurteilung*	
Entlassstatus (AU, AF, Stufe)	63,2 % AU
	35,6 % AF
	1,2 % Stufe
Leistungsfähigkeit im Bezugsberuf	61,5 % > 6 h/Tag
	11,7 % 3–6 h/Tag
	26,7 % < 3 h/Tag
Allgemeiner Arbeitsmarkt	71,6 % > 6 h/Tag
	10,4 % 3–6 h/Tag
	17,9 % < 3 h/Tag

*AF* arbeitsfähig, *AU* arbeitsunfähig, *Stufe* stufenweise Wiedereingliederung

### Häufigkeit der AEM-Reaktionsmuster bei Aufnahme und bei Entlassung

Von insgesamt *N* = 478 PCS-Patienten zu Reha-Beginn konnten insgesamt 67,7 % der Patienten dem Durchhaltemuster zugeordnet werden, wobei mit 57.7 % der größere Anteil dysfunktionale Distress-endurance-Formen aufwies; bei 10,0 % waren es Eustress-endurance-Muster. Bei 24,1 % lag mit „fear-avoidance“ ein weiteres dysfunktionales Muster vor. Das adaptive Muster war in dieser hoch chronifizierten Stichprobe nur mit 8,2 % vertreten.

Bei Entlassung zeigten sich signifikante Veränderungen mit positiven Verschiebungen zu mehr funktionalen Verhaltensweisen: mit 17,0 % etwa 9 % mehr AR und mit 22,7 % etwa 13 % mehr „eustress-endurance“. Die Ergebnisse sind in Tab. 2 dargestellt.

**Tab. 2:** AEM-Reaktionsmuster bei Aufnahme und bei Entlassung

	Aufnahme, *n *(%)	Entlassung, *n *(%)	Änderung von Aufnahme zu Entlassung (%)	*p*
Gesamt *N*	478 (100 %)	383 (80,13 %)	–	–
„Fear-avoidance response“ (FAR)	114 (24,1 %)	74 (19,3 %)	−4,8	0,001
„Distress-endurance response“ (DER)	276 (57,7 %)	157 (41,0 %)	−6,7	0,000
„Eustress-endurance response“ (EER)	48 (10,0 %)	87 (22,7 %)	+12,7	0,000
„Adaptive response“ (AR)	39 (8,2 %)	65 (17,0 %)	+8,83	0,000

*AEM* Avoidance-endurance-Modell

### Häufigkeit der AEM-Reaktionsmuster nach Rehabilitationsindikation

In allen Reha-Indikationen lag bei Aufnahme am häufigsten DER vor (68,6 % in der Psychosomatik, 53,8 % in der Neurologie, 63,3 % in der Pneumologie und 55,3% in der dualen Reha), die Indikationen unterscheiden sich hinsichtlich der Verteilung des DER-Musters nicht voneinander. Bei Entlassung unterschied sich die Pneumologie mit signifikant weniger FAR-Mustern von den anderen Indikationen (*p* = 0,046). Die Ergebnisse sind in Tab. 3 dargestellt.

**Tab. 3:** AEM-Reaktionsmuster bei Aufnahme zur Reha (T1) sowie zur Entlassung (T2), getrennt für die verschiedenen Reha-Indikationen

Reha-Indikationen	AEM-Muster							
	*FAR*		*DER*		*EER*		*AR*	
	T1	T2	T1	T2	T1	T2	T1	T2
*Psychosomatik*								
Aufnahme (*n* = 86)	18,6 %	–	68,6 %	–	9,3 %	–	1,4 %	–
Entlassung (*n* = 76)	–	14,5 %	–	48,7 %	–	21,1 %	–	15,8 %
*Neurologie*								
Aufnahme (*n* = 240)	27,5 %	–	53,8 %	–	8,8 %	–	10,0 %	–
Entlassung (*n* = 159)	–	25,2 %	–	40,9 %	–	18,9 %	–	15,1 %
*Pneumologie*								
Aufnahme (*n* = 49)	14,3 %	–	63,3 %	–	14,3 %	–	8,2 %	–
Entlassung (*n* = 46)	–	15,2 %	–	41,3 %	–	23,9 %	–	19,6 %
*Duale Reha*								
Aufnahme (*n* = 167)	25,2 %	–	55,3 %	–	11,7 %	–	7,8 %	–
Entlassung (*n* = 166)	–	15,7 %	–	35,3 %	–	29,4 %	–	19,6 %
*p*	0,217	0,056	0,217	0,229	0,207	0,999	0,017	0,078

*p* bezieht sich auf die Unterschiede zwischen den Indikationen zum jeweiligen Messzeitpunkt

*AEM* Avoidance-endurance-Modell, *AR* „adaptive response“, *DER* „distress-endurance response“, *EER* „eustress-endurance response“, *FAR* „fear-avoidance response“, *Reha* Rehabilitation

### Veränderung der AEM-Reaktionsmuster über den Rehabilitationsverlauf

Wie bereits beschrieben, lassen sich Veränderungen im Krankheitsverlauf über die Reha hinweg feststellen. In der bei Aufnahme vorliegenden Gruppe der Fear-avoidance-Typen blieben 36,4 % bei ihrem ängstlichen Vermeidungsverhalten, 19,3 % wechselten zu AR. Die restlichen FAR-PCS-Patienten veränderten ihr Verhaltensmuster zu 44,3 % zu Durchhaltemustern, wobei jeweils hälftig „distress“ und „eustress endurance“ vorlagen.

Die Subgruppe der Distress-endurance-Typen blieb hauptsächlich bei Durchhalteverhalten (78,9 %), wobei 30,0 % in ihrer Stimmung weg von „distress“ hin zu „eustress“ wechselten. Der Wechsel zu adaptiven Verhaltensweisen fiel ungefähr hälftig geringer aus als bei FAR.

Der Eustress-endurance-Typ blieb zu 25,0 % bei seinem positiv eingestelltem Durchhalten, 22,5 % wechselten zu adaptiven Verhaltensweisen (AR) und 25,0 % zu „distress-endurance“. FAR war mit 15,0 % bei EER etwas häufiger als bei DER.

**Abb. 1:**
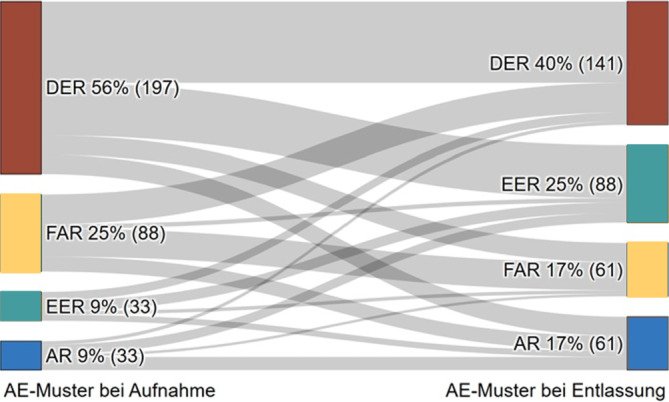
Veränderungen der AEM-Reaktionsmuster über den Rehabilitationsverlauf. *AEM* Avoidance-endurance-Modell, *AEQ* Avoidance-Endurance Questionnaire, *AR* „adaptive response“, *DER* „distress-endurance response“, *EER* „eustress-endurance response“, *FAR* „fear-avoidance response“, *T1* Zeitpunkt 1, *T2* Zeitpunkt 2

PCS-Patienten mit AR blieben ca. zur Hälfte (45,5 %) bei ihrem adaptiven Verhalten oder wechselten zu positiv eingestelltem Durchhalten (33,3 %). Insgesamt 21,2 % der PCS-Patienten, die sich zuvor adaptiv verhalten hatten, wechselten jedoch auch zu dysfunktionalen Verhaltensweisen wie FAR (9,1 %) und DER (12,1 %). Die Ergebnisse sind in Tab. 4 und Abb. 1 dargestellt.

**Tab. 4:** AEM-Reaktionsmuster. Änderung innerhalb der Subgruppe

*Subgruppe 100* *% FAR*
→ 36,4 % FAR
→ 38,6 % DER
→ 5,7 % EER
→ 19,3 % AR
*Subgruppe 100* *% DER*
→ 10,5 % FAR
→ 48,9 % DER
→ 30,0 % EER
→ 10,5 % AR
*Subgruppe 100* *% EER*
→ 15,0 % FAR
→ 25,0 % DER
→ 37,5 % EER
→ 22,5 % AR
*Subgruppe 100* *% AR*
→ 9,1 % FAR
→ 12,1 % DER
→ 33,3 % EER
→ 45,5 % AR

*AEM* Avoidance-endurance-Modell, *AR* „adaptive response“, *DER* „distress-endurance response“, *EER* „eustress-endurance response“, *FAR* „fear-avoidance response“

## Diskussion

Die hier durchgeführte Studie ist nach unserem Wissen die erste, die die Häufigkeit der unterschiedlichen Avoidance-endurance-Muster bei einer großen Stichprobe von PCS-Patienten und die Veränderbarkeit dieser Muster im Verlauf einer stationären Reha untersucht. Dass neben biologischen Faktoren auch psychische, verhaltensbezogene und soziale Aspekte eine entscheidende Rolle spielen, ist nach Stand der aktuellen Forschung zur Genese und Aufrechterhaltung chronischer Erkrankungen naheliegend [1, 2, 26]. Die bisherigen klinischen Erfahrungen mit PCS-Patienten lassen annehmen, dass sowohl ängstliches Vermeiden als auch Durchhalteverhalten fehlangepasste Reaktionen auf die mannigfaltige Symptomatik sind; diese Verhaltensmuster lassen sich auch in unseren Daten nachweisen.

Die hier untersuchte Stichprobe von *N* = 479 PCS-Patienten setzte sich vor allem aus Patienten mit einem Durchhaltemuster zusammen (67,7 %). Von diesen zeigte die Mehrheit mit 57,7 % vor Reha-Beginn Distress-endurance-Reaktionen, 10 % ein Eustress-endurance-Muster. Mit 24,1 % lag mit dem Angst-Vermeidungs-Verhalten (FAR) zu Reha-Beginn ein weiteres dysfunktionales Bewältigungsmuster vor. Lediglich 8,2 % wiesen ein adaptives Muster auf. Diese geringe Rate an adaptivem Verhalten liegt damit noch unter den Angaben für Patienten mit chronischen Schmerzen in der tertiären Versorgung [17, 21] und bestätigt die hohe Rate an dysfunktionalem Krankheitsverhalten bei hoch chronifizierten Patienten. Eine Studie zu chronischen Schmerzen von Hochleistungssportlern konnte zeigen, dass in dieser Patientengruppe vor allem das durchhaltende Verhalten der Eustress- und Distress-endurance-Reaktion mit 67,7 % vorherrschte, wobei EER häufiger (57,7 %) auftrat, DER jedoch zu einer höheren Beeinträchtigung führte [17]. Die hier untersuchten PCS-Patienten wiesen zwar ebenso wie die Hochleistungssportler mit chronischen Schmerzen vor allem Durchhalteverhalten auf, sie unterschieden sich jedoch hinsichtlich ihrer Stimmungslage; PCS-Patienten reagierten häufiger mit „distress-endurance“. Bei Nichtsportlern mit chronischen Rückenschmerzen in der Tertiärversorgung zeigten zwischen 58 % [21] und 62% [27] ein Durchhaltemuster, allerdings mit Überwiegen der Distress-endurance-Komponente; dies deckt sich mit unseren Ergebnissen der PCS-Patienten. Auch in einer Stichprobe von Patienten mit akuten/subakuten Rückenschmerzen überwogen Durchhaltemuster mit 19,2 und 16,4 % EER gegenüber 9,6 % FAR, allerdings war hier die Gruppe der adaptiven Muster deutlich stärker vertreten (54,8 % AR). Das AEM wurde auch an Patienten mit leichten bis moderaten Gehirnerschütterungssymptomen evaluiert [18], die noch Wochen danach an Symptomen wie Kopfschmerzen, Übelkeit und Müdigkeit litten. Es konnte hierbei insbesondere das Vorliegen von FAR nachgewiesen werden. Je nach Schweregrad der Symptome moderierte Katastrophisieren das Vermeidungsverhalten.

Es erscheint lohnend, Reha‑Maßnahmen hinsichtlich kognitiv-behavioraler Programme zu optimieren

Bis zum Ende der Reha veränderten 19,3 % der hier untersuchten Patienten mit einem FAR-Muster ihr Verhalten hin zu einer adaptiven Verarbeitung, bei Patienten mit EER waren es 22,5 % und bei DER lediglich 10,5 %. Zumal das dysfunktionale Muster DER bei fast jedem zweiten PCS-Patienten vorliegt, ist die sehr geringe Änderungsrate beachtlich. Inwiefern die hohe Beharrungstendenz mit Depressivität oder der Komponente des Durchhalteverhaltens zusammenhängt, bleibt weiterer Forschung vorbehalten. Im Rahmen der Schmerzforschung zum AEM wird angenommen, dass die ausgeprägte Neigung zur Gedankenunterdrückung bei DER-Patienten, die nur unregelmäßig zu einer erfolgreichen Unterdrückung von Schmerzen führt, mit häufigen Misserfolgserlebnissen und erhöhter Depressivität einhergeht, sodass das depressiv-suppressive Verarbeitungsmuster operanten Mechanismen der intermittierenden Verstärkung unterliegen könnte, was bekanntlich zu sehr zeitstabilem, habituellem Erleben und Verhalten führt [28]. Denkbar wäre zudem die Beteiligung einer Form des negativen Perfektionismus, für dessen Aufrechterhaltung ebenfalls Prozesse der intermittierenden negativen Verstärkung angenommen werden [19, 29]. Die Rate der Verbesserung bei FAR-Patienten ist vergleichbar zu den 30 % in der Studie von Cane et al. [22] bei Patienten mit chronischem Schmerz, das dysfunktionale Muster einer suppressiven Verarbeitung (vor allem DER) scheint allerdings gerade bei Patienten mit PCS wenig modifizierbar. Von den wenigen Patienten mit einem adaptiven Muster vor Reha blieben zudem lediglich 33,3 % konstant, 21,2 % entwickelten sich mutmaßlich dysfunktional. Diese Befunde machen deutlich, dass es lohnend erscheint, bestehende Reha-Maßnahmen hinsichtlich individuell indizierter kognitiv-behavioraler Programme zu optimieren.

Zusammengenommen lag bei etwa 60,3 % zu Reha-Ende ein Verhaltensmuster mit negativer Affektivität vor („distress-endurance“ oder „fear-avoidance“). EER-Reaktionen nahmen um 12,7 % zu. Bei diesem Muster ist unklar, ob es ähnlich wie bei der Schmerzchronifizierung als dysfunktional zu bewerten ist [16, 17]. Durchhalteverhalten im Sinne von positiver Neubewertung und Ablenkung kann als adaptive Reaktion bei Depression und Angst genutzt werden, die auch häufig mit Schmerz in Verbindung stehen. Studien legen jedoch nahe, dass die EER im Sinne des AEM bei Studien an Patienten mit chronischem Schmerz keine adaptive Form der Krankheitsbewältigung darstellt [27, 30]. Kurzfristig könnte EER bei akutem/subakutem Schmerz zu mehr Leistung und zu einem Stimmungsaufschwung führen. Zugleich könnten jedoch auch hohe Leistungsansprüche in Zusammenhang mit EER stehen, die langfristig zu mehr Schmerz führen könnten [21, 27, 31]. Es gibt erste Hinweise darauf, dass dieses Muster bei Fatigue im Rahmen eines PCS eine Zwischenstellung einnimmt oder möglicherweise sogar adaptiv wirken kann [20, 32]. Hierbei wäre künftig zu untersuchen, ob EER-PCS-Patienten eher ein adaptives Pacing betreiben oder doch zu Überlastung neigen, die kurzfristig durch positive Neubewertung und Ablenkung überdeckt wird.

Unsere Studie zeigt, dass etwa 68 % der PCS-Patienten ein dysfunktionales Bewältigungsmuster vom Distress-endurance- oder vom Fear-avoidance-Typ aufweisen, wobei fast die Hälfte Distress-endurance-Typen bzw. dysfunktionale Durchhalter sind. Darüber hinaus zeigen unserer Daten, dass sich über den Reha-Verlauf Krankheitsverhalten ändert, die Beharrungstendenzen in den unterschiedlichen Mustern jedoch unterschiedlich ausfallen (DER: 48,9 % Beharrung, FAR: 36,4 % Beharrung). Auch die Chance auf einen Wechsel hin zum funktionalen Typ ist zwischen FAR, DER und EER ungleich (Wechsel zum funktionalen Typ: FAR 19,3 %, DER 10,5 %, EER 22,5 %).

Schmerzen nach einer akuten SARS-CoV-2-Infektion (Post-COVID-Schmerzen) entwickeln sich zu einem neuen Problem des Gesundheitswesens, werden aber nach wie vor unterschätzt und höchstwahrscheinlich nicht ausreichend behandelt, weil das Phänomen nicht erkannt wird und die zugrunde liegenden Schmerzmechanismen nicht bekannt sind. Es gibt keine ausreichenden Belege für einen bestimmten Behandlungsansatz zur Behandlung von PCS-Schmerzen. Klinisch wird eine große Variabilität beim Ansprechen der Patienten auf Standardschmerzbehandlungen beobachtet, was zu Forderungen nach einem personalisierten, maßgeschneiderten Ansatz für die Behandlung von Patienten mit chronischen PCS-Schmerzen geführt hat (Stichwort „Präzisionsschmerzmedizin“). Es gibt Vorschläge, dass die Unterteilung des PCS-Schmerzes in nozizeptive, neuropathische, noziplastische oder gemischte Typen der erste Schritt zu einer besseren Planung von Behandlungsprogrammen ist [33]. Ferner wird empfohlen, neben Faktoren wie Geschlecht und somatischen Komorbiditäten auch das Vorhandensein von psychischen Störungen in eine Therapie einzubeziehen [34]. Hier wird vorgeschlagen, neben einer multimodalen pharmakologischen Behandlung auch nichtpharmakologische Maßnahmen zu berücksichtigen, die auf emotionale/kognitive Aspekte (das heißt psychologische und/oder Bewältigungsstrategien), auf mit zentraler Sensibilisierung verbundene Mechanismen (das heißt schmerzneurowissenschaftliche Aufklärung), auf Bewegungsprogramme sowie auf Maßnahmen zur Verbesserung des Lebensstils (beispielsweise Ernährungsberatung und Schlafmanagement) abzielen [34].

## Stärken und Limitationen

Die hier vorliegende Studie basiert auf einer großen Stichprobe von *N* = 478 und untersucht unseres Wissens erstmalig die Häufigkeit und Veränderbarkeit der AEM-Reaktionsmuster bei stationären PCS-Reha-Patienten. Einige Limitationen dieser Studie sollen jedoch benannt werden. Bei den eingeschlossenen PCS-Patienten handelt es sich um eine bereits stark chronifizierte Stichprobe, sodass die Ergebnisse, insbesondere die Häufigkeitsverteilung der AEM-Muster, nur auf Settings mit vergleichbar chronifizierten Patienten (beispielsweise Reha) übertragen werden können. Vermutlich wäre in einer weniger chronifizierten und belasteten Stichprobe der Anteil an AR und EER höher. Zudem ist noch zu prüfen, inwiefern die vielen Wechsel zwischen den verschiedenen AEM-Mustern auch eine Problematik der Test-Retest-Reliabilität sein könnten oder ob viele Patienten ohnehin schon in Grenzbereichen zwischen mehreren Typen angesiedelt waren. Ein Großteil der PCS-Patienten ist bis zum Reha-Beginn mehr als ein Jahr erkrankt und weist damit bereits eine starke Chronifizierung auf, sodass unsere Ergebnisse vor diesem Hintergrund zu betrachten sind. Studien zu potenziellen Einflussfaktoren der AEM-Reaktionsmuster auf die Symptomatik sind notwendig, um Hinweise zur künftigen Schwerpunktsetzung der Therapien zu erhalten.

## Fazit für die Praxis

- Die Ergebnisse dieser Studie zeigen, dass etwa 81,8 % der Patienten mit Post-COVID-Syndrom (PCS) ein eindeutig dysfunktionales Krankheitsverhalten aufweisen, wobei hiervon 57,7 % dysfunktionale Durchhalter (mit „distress-endurance response“) sind.
- Über den Rehabilitationsverlauf verschieben sich insgesamt die Verhaltensmuster um 8,8 % hin zur „adaptive response“ und um ebenfalls etwa 13 % zum Eustress-endurance-Muster, wobei noch nicht klar ist, ob dieses Muster bei Fatigue im Rahmen eines PCS eine Zwischenstellung einnimmt oder möglicherweise sogar adaptiv wirken kann.
- Innerhalb der einzelnen Muster des Avoidance-endurance-Modells (AEM) können sich dysfunktionale Verhaltensweisen zugunsten funktionaler verändern, aber auch umgekehrt funktionale Muster zu dysfunktionalen.
- Die Bewältigungsstile nach dem AEM konnten bei PCS-Patienten nachgewiesen werden, und unsere Daten stützen die Hypothese, dass das Durchhaltemuster bei PCS besonders bedeutsam ist. Die Veränderungen der Muster über den Rehabilitationsverlauf zeigen aber auch, dass schon zu Beginn auf funktionale Verhaltensmuster therapeutisch achtgegeben werden muss, um einer Verschiebung zu dysfunktionalen Mustern vorzubeugen.

## Supplementary Information

Deskriptive Statistiken der Gesamtstichprobe und der einzelnen Indikationen

## Data Availability

Da es sich bei den Daten um Versichertendaten der Deutschen Rentenversicherung handelt und ein strenger Datenschutz besteht, sind diese Daten nicht öffentlich zugänglich, können aber auf begründeten Antrag vom jeweiligen Autor in einer für personenbezogene Daten angepassten Form zur Verfügung gestellt werden.

## Abkürzungen

ADS: „Anxiety/depression scale“ (Angst-Depressions-Skala)
AEM: Avoidance-endurance-Modell
APAS: „Avoidance of physical activities scale“ (Skala für Meiden körperlicher Aktivitäten)
AR: „Adaptive response“
ASAS: „Avoidance of social activities scale“ (Skala für Meiden sozialer Aktivitäten)
BDI-II: Beck Depression Inventory-II
BES: „Behavioral endurance scale“
COVID: „Coronavirus disease“
COVID-19: „Coronavirus disease 2019“
CTS: „Catastrophizing scale“ (Katastrophisierungsskala)
DER: „Distress-endurance response“
EER: „Eustress-endurance response“
FAR: „Fear-avoidance response“
HDS: „Humor/distraction scale“ (Humor-Ablenkungs-Skala)
HHS: „Helplessness/hopelessness scale“ (Skala für Hilf‑/Hoffnungslosigkeit)
PCS: Post-COVID-Syndrom
PHQ‑9: Patient Health Questionnaire‑9
PMS: „Positive mood scale“ (Skala für positive Stimmung trotz Fatigue)
PoCoRe: Post-COVID-Rehabilitation
PPS: „Pain persistence scale“
SARS-CoV‑2: „Severe acute respiratory syndrome coronavirus type 2“
TSS: „Thought suppression scale“ (Gedankenunterdrückungsskala)
