# Supplementary material for: Importance of avoidance and endurance in post-COVID syndrome: Can dysfunctional patterns be changed?
Source: Schmerz. 2025 Jun 20;39(5):329–38. [Article in German] doi: 10.1007/s00482-025-00887-5 (PMC13035739; doi:10.1007/s00482-025-00887-5)
Supplement: Supplementary file 1 — Deskriptive Statistiken der Gesamtstichprobe und der einzelnen Indikationen [file 482_2025_887_MOESM1_ESM.pdf]

Online-Zusatzmaterial

Tabelle S1 Deskriptive Statistiken der Gesamtstichprobe und der einzelnen Indikationen

	Gesamtstichprobe	Duale Reha	Psychosomatik	Neurologie	Pneumologie	p
Demographische Eigenschaften						
Geschlecht (% weiblich)	71.5 % ♀	71.4 % ♀	67.0 % ♀	68.1 % ♀	77.0%♀	<.001
Alter ♀	48.30 (10.92)	51.77 (8.50)	41.84 (8.48)	48.07 (10.72)	47.10 (10.90)	<.001
Berufliche Merkmale						
Arbeits(un)fähigkeit bei Aufnahme	60.3 % AU<br>39.5 % AF	61.1 % AU<br>38.9 % AF	68.1 % AU<br>31.9 % AF	61.9 % AU<br>37.7 % AF	49.4% AU<br>50.6% AF	<.001
Zeiten der Arbeitsunfähigkeit vor Beginn der Rehabilitation	12.9 % <3 Monate<br>8.0 % 3-6 Monate<br>78.8 % >6 Monate	23.1 % <3 Monate<br>3.4 % 3-6 Monate<br>73.5 % >6 Monate	23.7 % <3 Monate<br>12.2 % 3–6 Monate<br>64.0 % >6 Monate	13.0 % <3 Monate<br>8.1 % 3–6 Monate<br>78.6 % >6 Monate	15.2 % <3 Monate<br>8.7 % 3–6 Monate<br>76.1 % >6 Monate	<.001

*Anmerkung. AU = arbeitsunfähig, AF = arbeitsfähig
